# Spanish ecological battleground: population structure of two invasive fungi, *Cryphonectria parasitica* and *Fusarium circinatum*

**DOI:** 10.3389/fpls.2023.1310254

**Published:** 2023-12-21

**Authors:** Farooq Ahmad, Julio Javier Diez

**Affiliations:** ^1^ Department of Plant Production and Forest Resources, University of Valladolid, Palencia, Spain; ^2^ Sustainable Forest Management Research Institute, University of Valladolid and INIA, Palencia, Spain

**Keywords:** genetic diversity, invasive plant pathogens, chestnut blight fungus, pine pitch canker (PPC), ITS sequence analysis, mating type (MAT) alleles, vegetative compatibility group (VCG), population genetics

## Abstract

**Introduction:**

Invasive fungi distributed worldwide through globalization have caused devastating diseases in different forests, causing economic and ecologic disturbances. Two such invasive species are *Cryphonectria parasitica* and *Fusarium circinatum*, which were introduced to Europe from North America, separated temporally: *C. parasitica* was introduced about nine decades ago, whereas *F. circinatum* was introduced around two decades ago. As *C. parasitica* had a longer time to undergo genetic changes, we hypothesized that it has higher genetic diversity than the recently introduced *F. circinatum* in Spain. In addition, we studied the genetic characterization of both fungi present in similar ecological conditions in Northern Spain with the aim of providing data for biocontrol measures.

**Methods:**

Molecular genetic markers were used to test these hypotheses, including mating type and DNA sequencing of internal transcribed spacer (ITS) regions. In addition, we used vegetative compatibility (VC) type markers in *C. parasitica* as the information about VC type is essential to apply biocontrol against the fungus.

**Results and discussion:**

All the isolates of *C. parasitica* from the studied area belonged to only one VC type (EU-1) and one mating type (MAT-2). However, three distinct haplotypes of *C. parasitica* were identified through ITS sequencing, showing that multiple introductions might have happened to Cantabria. Among *F. circinatum*, no diversity was observed in ITS and MAT loci in the studied area but isolates from other Spanish regions showed the presence of both mating types. Overall, *C. parasitica* had higher genetic diversity than *F. circinatum*, despite both organisms appearing to reproduce clonally. This study helped understand the invasion patterns of *C. parasitica* and *F. circinatum* in northern Spain and will be useful in applying biocontrol measures against both pathogens.

## 1 Introduction

Advancements in high-throughput sequencing have made it possible to observe that plants host a range of fungi (Schlaeppi and Bulgarelli, 2015). Most of these fungal species have co-evolved with their hosts and form a mutualistic relationship with hosts of their native range (Sieber, 2007). However, some fungal species become pathogenic to plants of exotic places that did not co-evolve with the fungus, for example, when they move to different continents. The introduction of such invasive fungi has caused several epidemics in the past, threatening native plant species. The introduction of *Cryphonectria parasitica* and *Fusarium circinatum* is one of many such examples (Rigling and Prospero, 2018; Drenkhan et al., 2020). Globalization has caused a dramatic increase in the number of invasions, especially in the past 50 years (Seebens et al., 2017).

When a fungus is introduced to another continent, it meets native hosts with little or no protection against the pathogen. This can be devastating for native plant species and forests; for example, the fungus *Cryphonectria parasitica* killed around 4 billion chestnut trees after it was introduced to North America from East Asia (Ghelardini et al., 2017). *Ophiostoma* spp. is another invasive fungus that killed around 40 million elm trees in the UK alone (Brasier, 2008). *Fusarium circinatum* is another invasive fungus threatening the *Pinus* species due to its broad host range (Drenkhan et al., 2020). The introductions of invasive fungal species have caused significant economic and ecological consequences, but their invasion histories remain less explored than invasive plants and animals. In recent years, the number of forest pathogens invasions has increased due to a significant increase in global trade (Santini et al., 2013; Maxwell et al., 2014; Seebens et al., 2017; Antonelli et al., 2022).

*Cryphonectria parasitica* is an Ascomycota fungus that originated from East Asia, likely from China or Japan (Milgroom et al., 1996; Liu and Milgroom, 2007). It causes chestnut blight disease and manifests severe symptoms in European and American chestnut trees, whereas the symptoms in Asian chestnuts are mild to none (Rigling and Prospero, 2018). The fungus was introduced to Europe in the 1930s from North America in several European populations of chestnuts (Milgroom et al., 1996). *Cryphonectria hypovirus 1* (CHV-1) containing strains of *C. parasitica* are less virulent, so they are used as biocontrol (Nuss, 2005). *Cryphonectria- Hypovirus* pathosystem is a textbook example of biocontrol through mycoviruses. CHV-1 (naturally or introduced artificially) has successfully controlled the disease and saved the trees from death in many European populations (Jeger et al., 2016; Rigling and Prospero, 2018). However, the disease continues to exist in all chestnut-growing European countries and threatens European chestnuts (EPPO, 2019). The biocontrol helped to limit the damage caused by the pathogen, but *C. parasitica* populations still co-exist with most of the European chestnuts. Nevertheless, many *C. parasitica* populations remained uncharacterized, so no control could be applied. These fungal populations not only threaten chestnut cultivation in their areas but can serve as a source of virulent (without CHV-1) strains in other populations where biocontrol is successfully applied (Ahmad and Baric, 2022a).

A high mortality rate has been observed across different chestnut populations in Europe in the last decades, caused by a combined effect of biotic stresses and climate change (Waldboth and Oberhuber, 2009; Ahmad and Baric, 2022a). Another reason for this high mortality could be the increasing genetic diversity of the pathogen in the European population (Ahmad and Baric, 2022a), especially the vegetative compatibility type (VC) diversity. The increase in genetic diversity is mainly due to sexual reproduction, as almost a century has passed since its introduction, signifying the successful establishment of the fungus in Europe (Ahmad and Baric, 2022a). Additional introduction events directly from North America and Asia have been detected that also caused an increase in the genetic diversity of the fungus (Dutech et al., 2012; Ahmad and Baric, 2022a). As a result, the genetic diversity in the North Italian populations has triplicated in the last 25 years (Ahmad and Baric, 2022a). In other countries also, genetic diversity increased, especially VC-type diversity; for example, in Croatia, the VC-type number increased from 18 to 26 (Krstin et al., 2008; Mlinarec et al., 2018), in Western Spain, from six to eleven (Montenegro et al., 2008; Zamora et al., 2012), in Germany, from five to thirteen (Peters et al., 2012; Peters et al., 2014), and in Great Britain, from eleven to twelve (Pérez-Sierra et al., 2019; Romon-Ochoa et al., 2021).

Another pathogen introduced to Europe recently is *F. circinatum*, which causes pine pitch canker. The fungus also belongs to the Ascomycota division and is pathogenic to different *Pinus* spp., especially *Pinus radiata* (Iturritxa et al., 2013). It is thought to have originated from North America (somewhere between Southern USA and Northern Mexico) but is currently present in 14 countries around the world and represents a threat to Pine species (Berbegal et al., 2013; Vettraino et al., 2018; Drenkhan et al., 2020).

Population genetic markers are used to study the invasion patterns and monitor the spread and establishment of invasive species (Le Roux and Wieczorek, 2009). Some markers are also useful to optimize biocontrol measures, such as vegetative incompatibility type loci (*vic*) that play a role in the successful spread of mycoviruses among different fungal strains. Mating type markers are useful to estimate the potential of sexual reproduction because many Ascomycota fungi, such as *C. parasitica* and *F. circinatum*, can reproduce both clonally and sexually. Internal Transcribed Spacer (ITS) region is also useful for investigating invasion history because of the abundance of ITS sequences in the online databases (Ahmad and Baric, 2022a).

*Cryphonectria parasitica* and *F. circinatum* are invasive alien species in Spain, but their introduction events are separated temporally. *Cryphonectria parasitica* was introduced in the 1930s and is now present in many Spanish areas (Bascón et al., 2014), whereas *F. circinatum* was first reported in 2005 (Landeras et al., 2005). Therefore, *C. parasitica* had a longer time to undergo genetic changes through mutation, recombination, and genetic drift. This provides perfect grounds to compare the genetic diversity of both invasive fungi in the region. Here, we hypothesized that *C. parasitica* populations have higher genetic diversity in Spain than *F. circinatum* because they are better established in the local populations. For this purpose, we used mating type markers and ITS sequencing analysis in Spain’s *C. parasitica* and *F. circinatum* populations. The second objective of this study was to provide data that can be used to optimize the biocontrol strategies of both species in the Autonomous Community of Cantabria. For this reason, we also studied vegetative compatibility types of *C. parasitica* populations present throughout the previously uncharacterized populations of Cantabria. Finally, we perform a sequence analysis of data downloaded from the nucleotide database (NCBI Genbank) to test our hypothesis.

## 2 Materials and methods

### 2.1 Survey, sampling, and isolation of fungal cultures

Field trips were carried out to chestnut tree populations throughout the Autonomous Community of Cantabria, Spain. Between August 2022 and February 2023, diseased trees showing characteristic symptoms of chestnut blight (yellow to orange canker on the trunk) were sampled throughout the region. Small rectangular bark pieces (2cmX10cm) were cut with the help of a chisel in the border areas between the canker and healthy bark. A total of 179 samples were collected from all over Cantabria, as shown in **Table 1**. Only one sample per tree was collected.

**Table 1:** Samples of *Cryphonectria parasitica* collected in this study at the Autonomous Community of Cantabria (Northern Spain).

Forest division	Municipality	Area	N Samples
1	Peñarrubia	La Rampa Roza	12
		Santa Catalina	
3	Tudanca	Sarceda	12
	Rionasa	Santa Agueda	11
		Rozadio	11
7	Arenas de Iguña	Castañera Los Llanes	13
	Bárcena Pie de Concha	–	16
	Cieza	–	9
	Los Corrales de Buelna	Elgedo	14
		Fresneda	4
9	Villafufre	–	21
10	Arredondo	Bustablado	9
		Las Lloverizas	10
		Las Rozas	4
		Río Asón	12
		Socueva	11
		Soncillo-Villar	10
Total samples			179

*Cryphonectria parasitica* was isolated through two methodologies: Incubation in humid chambers and direct isolation. All the bark samples were cut into two, surface cleaned with 3% hydrogen peroxide solution, washed with distilled-autoclaved water and incubated in the Potato Dextrose agar medium (PDA) for 7 to 20 days. If the *C. parasitica* was contaminated, the mycelia were transferred to a fresh PDA plate. Whenever purifying the fungal culture was impossible, the humid chamber extraction was performed according to the protocol (Ahmad and Baric, 2022a). Pure fungal isolates obtained from both methods were maintained on the Petri dishes containing PDA at room temperature.

*Fusarium circinatum* isolates were taken from the collection of Forest Pathology Lab (University of Valladolid) and fresh cultures of the isolates were prepared on PDA medium. The highest number of isolates (36) were from Cantabria because we wanted to know the variability of *F. circinatum* in Cantabria and compare it with the population structure of *C. parasitica*. However, isolates from other regions were also included to see if there was any genetic diversity of *F. circinatum* in Spain. A total of 67 isolates were used in this study to characterize the populations of *F. circinatum* in Spain (**Table 2**).

**Table 2:** Isolates of *Fusarium circinatum* used in this study.

Autonomous community	Municipality	N Samples
Asturias	–	5
Cantabria	Cabezón de la Sal	11
	Cabuérniga	1
	Castro Urdiales	2
	Comillas	3
	Mazcuerras	4
	Rionansa	6
	Santirude de Toranzo	5
	Villafufre	4
Galicia	–	7
Basque country	–	4
Castilla y León	–	15
Total	–	67

### 2.2 DNA extraction

For DNA extraction, the protocol described by Cassago et al. (2002) was followed with slight modifications as described: Purified fungal strains were grown in the PDA medium for 7-10 days. The mycelia were collected with a sterile pipette tip, avoiding collecting PDA and deposited in a 1.5 mL microcentrifuge tube containing 500 μL of a resuspension buffer. To this tube, four tungsten beads were added and shaken vigorously with a ball mill (TissueLyser, Retsch^®^), for two minutes and left on ice for one minute. To the solution, 150 μL of potassium acetate at pH 4.8 was added. The tube was mixed in a vortex and centrifuged at 15000Xg for two minutes. The supernatant was transferred to a different 1.5 mL microcentrifuge tube and 600 μL of isopropanol was added. After mixing the tube by inversion, it was centrifuged at 15000Xg for 10 minutes at 4°C. The DNA pellet was cleaned with 500 μL of ethanol (70%) and centrifuged at 16000Xg for 10 minutes. The 500 μL of ethanol is then removed with a pipette, taking great care not to collect the DNA pellet. The latter was allowed to air dry under laminar flow overnight. After this, the DNA was resuspended in 50 μL of TE-RNAs buffer. It was then stored for the short-term at 4°C or long-term at -20°C.

### 2.3 PCRs and gel electrophoresis

FastGene^®^ DNA Polymerase Taq polymerase (NIPPON Genetics Europe) was used to perform the Polymerase Chain Reactions (PCRs) in the following final concentrations: 1X buffer B, dNTPs mix 200uM, 1 U of DNA polymerase and 1 ul of DNA. The PCRs for vegetative compatibility types in *C. parasitica* were multiplexed according to the protocol designed by (Ahmad and Baric, 2022a). The PCRs of mating-type loci in *F. circinatum* were also multiplexed according to the published protocols (Wallace and Covert, 2000; Schweigkofler et al., 2004). The concentrations of each primer and their annealing temperatures are available in **Supplementary Table S1**. The PCR conditions were the following: initial denaturation at 95°C for 3 minutes, followed by 40 cycles of denaturation at 95°C for 30 sec, annealing at different temperatures depending on the primers (**Supplementary Table S1**), and extension at 72°C for 45 seconds. The final extension was done at 72°C for 5 minutes. PCR products were observed on 1.5% agarose gels after 40 min of separation at 80 Volts.

### 2.4 Purification of PCR products and sequencing

PCR products were purified through NucleoSpin Gel and PCR Clean−up kit (Macherey-Nagel, Germany), using the manufacturer’s protocol. The purified PCR products of ITS regions were sequenced through Stabvida, Portugal (https://www.stabvida.com) through Sanger Sequencing, using the feature you tube it/you plate it.

### 2.5 Data analysis

Isolates of *C. parasitica* were classified in EU vegetative compatibility types based on their allelic data, according to Cortesi and Milgroom (1998). Geneious Prime (version 2023.0.1) was used to manually check the sequences and align them using the option of MUSCLE alignment. The nucleotide sequences from the previous studies were downloaded from NCBI Genbank by the text “*Cryphonectria parasitica* internal transcribed spacer” and “*Fusarium circinatum* internal transcribed spacer”. A list of downloaded accession numbers and their respective locations are available in **Table S2** (*C. parasitica*) and **Table S3** (*F. circinatum*). All the sequences were downloaded and aligned using the same programs mentioned above. The sequences that showed a high number of polymorphisms were deleted from the list of downloaded sequences and a new alignment was performed. Mesquite was used to convert the Fasta alignment to.nex file type because this is the file that can be used by the other software we used. The information about each sequence was also recorded from the Genbank into a trait file. The alignment and trait files were then imported to POPART, version 1.7 (https://popart.maths.otago.ac.nz), and haplotype networks were made using the algorithm of the Median Joining Network. The phylogenetic tree was constructed using MEGAX, version 10.0.5. through the Neighbour Joining Algorithm.

## 3 Results

### 3.1 Isolation of *Cryphonectria parasitica*

Out of 179 samples, 122 pure isolates of *C. parasitica* could be obtained. The remaining samples resulted in no *C. parasitica* isolates or contaminated cultures, which could not be purified even after repeated attempts. Contaminated isolates were discarded after autoclaving and only pure isolates were used for further genotyping. Although we lost many cultures due to contamination, the purification rate was 68.15%.

### 3.2 Vegetative compatibility types of *C. parasitica* in Cantabria

All the pure isolates of *C. parasitica* were amplified through four different multiplex PCRs. Sizes of alleles that were observed on gel electrophoresis for each *vic* loci are shown in **Table 3**. The results from these PCRs showed that all the *vic* loci were monomorphic and no diversity was observed. One hundred and twenty-two isolates had allele 2 on *vic1a*, *vic2, vic4, vic6* and *vic7* loci. In contrast, allele 1 was present only on *vic3a* locus. Therefore, the genotype of all the isolates was 2212-22. This genotype belongs to the vegetative compatibility type EU-1, as suggested by Cortesi and Milgroom (1998). No other vegetative compatibility (VC) type of *C. parasitica* was detected in our study from Cantabria.

**Table 3:** Vegetative incompatibility loci (*vic*) of *Cryphonectria parasitica* and the number of isolates that resulted into each allele.

Locus	Allele 1	Allele 2
*vic1a*	0	122
*vic2*	0	122
*vic3a*	122	0
*vic4*	0	122
*vic6*	0	122
*vic7*	0	122

### 3.3 Variability of *Fusarium circinatum* based on the DNA sequence

The amplified PCR products of the ITS region of *F. circinatum* showed an amplicon of approximately 600 base pairs (bp) on the Agarose gel. After trimming, 463bp long sequences were achieved. The ITS sequences from all *F. circinatum* strains were monomorphic in our study and showed no variability. When the haplotype was blasted on the Genbank (NCBI), it was 100% identical with 100% query coverage to many *F. circinatum* sequences, such as accession codes MT464451 and MN326463. The haplotype of *F. circinatum* found in this study was deposited to the Genbank with accession number OR286415.

### 3.4 Variability of *Cryphonectria parasitica* based on the DNA sequence

**Figure 1:**
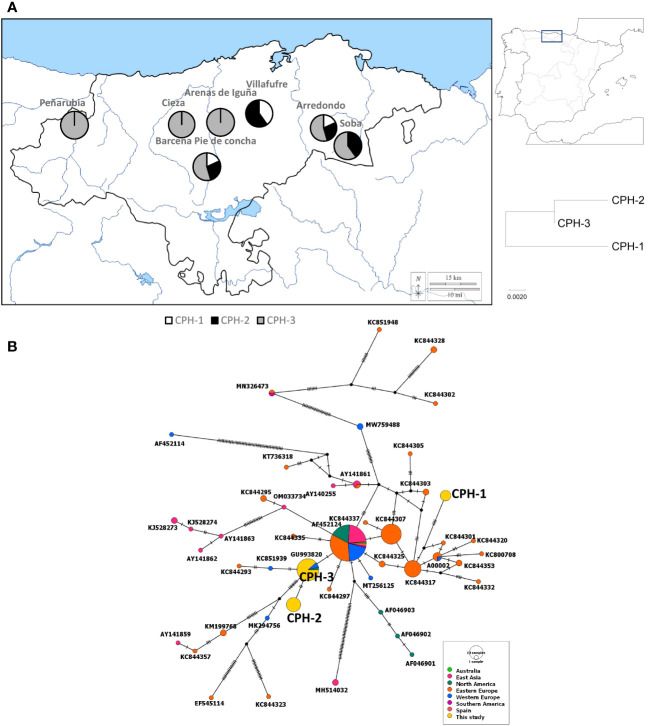
Genetic diversity of *Cryphonectria parasitica* through the sequencing of internal transcribed spacer region. **(A)** The occurrence of different haplotypes in different municipalities of Cantabria (left). The code CPH refers to *C. parasitica* haplotype. A zoomed-out map of Spain where the studied area is marked (right-above). A phylogenetic tree showing the evolutionary relationship based on the genetic distances between the haplotypes found in this study (right-below). **(B)** A median joining network shows the relationship between different haplotypes of the fungus found in this study and previous studies. The diameter of each circle represents the frequency of the haplotype whereas the colour represents the sampling location of the isolate.

The amplified PCR products of the ITS region of *C. parasitica* showed an amplicon of approximately 650 base pairs on the Agarose gel. After trimming, 598bp long sequences were achieved that were used to make haplotypes. Sequence analysis showed 3 different haplotypes among all *C. parasitica* isolates. Haplotype 3 of *Cryphonectria parasitica* (CPH-3) was the most dominant in the region, whereas CPH-1 was the least dominant haplotype of *C. parasitica* present in the studied area. CPH-3, the most dominant haplotype, was found in all municipalities except Villafufre (**Figure 1**). CPH-1 was reported in the Arredondo, Villafufre and Barcena Pie de Concha isolates. CPH-2 was reported in the isolates from Arredondo, Villafufre, Barcena Pie de Concha and Soba. Overall, Central and Eastern Cantabria municipalities had a diverse haplotype group, whereas the Western Cantabria had *C. parasitica* belonging to only one haplotype (CPH-3). Three haplotypes of *C. parasitica* found in this study were deposited to the Genbank with accession numbers OR272518, OR272519, and OR272520, respectively.

### 3.5 ITS sequence analysis of *Cryphonectria parasitica* and *Fusarium circinatum*

**Figure 2:**
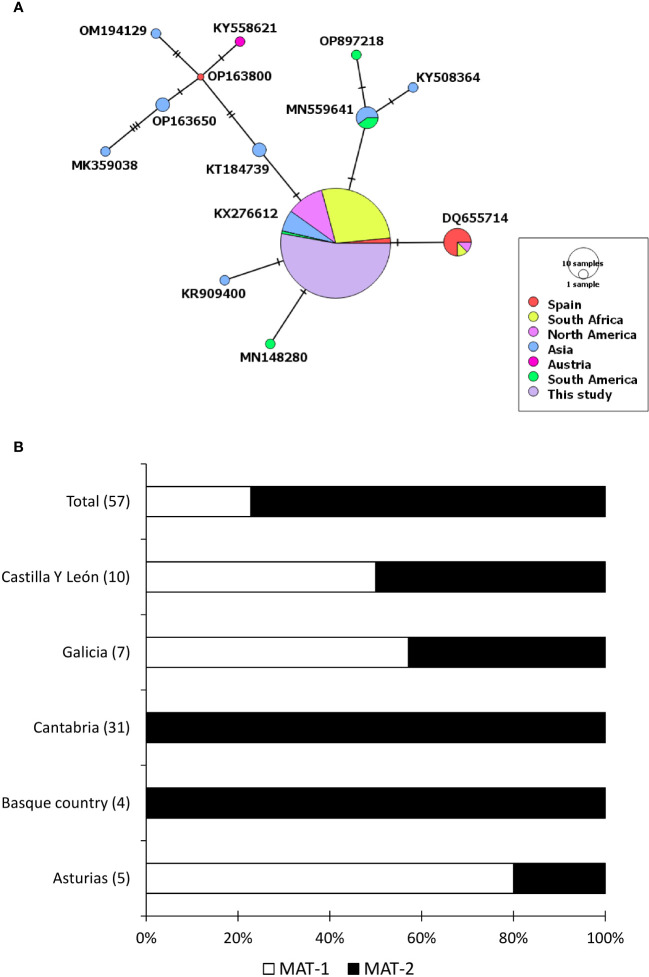
Genetic diversity of *Fusarium circinatum* found in this study. **(A)** A median joining network that shows different haplotypes of the fungus found through sequencing of internal transcribed spacer region. The diameter of each circle represents the frequency of the haplotype whereas the colour represents the sampling location of the isolate. **(B)** Percentage of isolates that belongs to a particular mating type found in different autonomous communities of Spain. Number in the parenthesis show the total number of samples.

When analysed with sequences downloaded from Genbank, the haplotype of *F. circinatum* that was found in our study was the most common globally and found in other countries such as South Africa, Mexico, the United States of America, Brazil, South Korea and India (**Figure 2**). Overall, we found that *F. circinatum* strains have low genetic diversity in Spain and globally compared to *C. parasitica*. *Cryphonectria parasitica*, on the other hand, showed a high genetic diversity around the world and in Spain. Among the three *C. parasitica* haplotypes, CPH-3 has already been reported in North America, Western Europe, and East Asia. However, CPH-1 and CPH-2 were unique to this region and were found for the first time. CPH-2 had only 2 different substitutions when compared with CPH-3. Both CPH-2 and CPH-3 were similar to the haplotype that is the most dominant in the world and is found in different regions (**Figure 1**). However, CPH-1 was very different and was more similar to the haplotypes previously reported in East Asia.

### 3.6 MAT of *Fusarium circinatum* was more diverse than *C. parasitica*

PCR amplification of the mating type (MAT) 2 allele in *Cryphonectria parasitica* showed an amplicon of around 1700 bp, whereas mating type 1 was not amplified in any PCR. Among 122 isolates, 115 were amplified, whereas MAT locus of seven isolates could not be amplified. Therefore, it was concluded that 115 isolates of *C. parasitica* in the present study belonged to MAT-2, and MAT-1 was not detected in the studied area. Multiplex PCR of *F. circinatum* mating types showed either 380 bp or 190 bp amplicon on Agarose gel, showing MAT-1 and 2, respectively. Among 67 isolates of *F. circinatum*, 13 belonged to MAT-1 and 44 belonged to MAT-2. The remaining ten isolates could not be assigned to any mating type. Six isolates showed no amplification product, whereas 4 isolates showed both amplification products on the gel.

Mating types of *F. circinatum* were differently distributed in different regions, with an overall ratio between MAT-1 and MAT-2 of approximately 1:3. The isolates from Cantabria and Basque country belonged to MAT-2 only. In contrast, isolates from Galicia, Castilla y León and Asturias belonged to both mating types (**Figure 2**). Castilla Y León was the only region where the mating type ratio was 1:1, whereas the ratio was also closer to 1:1 in Galicia.

## 4 Discussion

Chestnut and Pine trees are essential to the Spanish landscape and provide several social and economic services. Devastating diseases such as chestnut blight and pine pitch canker represent the biggest threat to both species, respectively (Rigling and Prospero, 2018; Drenkhan et al., 2020). We isolated *C. parasitica* strains directly from the symptomatic trees for genetic characterization. Although we lost many cultures of *C. parasitica* due to contamination, the purification rate was higher than in a previous study (Ahmad and Baric, 2022a). One reason for the lower isolation rates of the fungus is that isolating fungi from wood material is much harder than isolating from other plant parts, i.e., leaves (Kovalchuk et al., 2018; Diez-Hermano et al., 2022). Overall, low genetic diversity of both pathogens was found in Northern Spain as compared to other European countries. We also showed that they are not sexually active in many Spanish populations. Only one VC type of *C. parasitica* was found in the studied area, showing that it should be easy for the mycoviruses to spread and develop themselves due to no barrier posed by hyphal anastomosis. Interestingly, three different haplotypes of *C. parasitica* were observed through ITS sequencing, showing that the populations are not as monomorphic as they appeared with *vic* and MAT markers. Overall, a higher genetic diversity of *C. parasitica* than *F. circinatum* was found in Cantabria (Northern Spain). This is the first study conducted on *C. parasitica* populations in Cantabria, so it significantly contributes to the disease management efforts.

In *C. parasitica* populations, EU-1 was the only VC type found; the same VC type is not only present in Spain but is the most diverse VC type in many Spanish communities. In Asturias, for example, around 95% of the isolates belonged to EU-1 (González-Varela et al., 2011). In the province of León also, approximately 80% of the isolates belonged to EU-1 and the same mating type, MAT-1 (Montenegro et al., 2008; Zamora et al., 2012). Asturias and Leon are neighboring regions of Cantabria, so they seem to have similar populations. This leads us to two hypotheses: 1) Cantabrian populations of *C. parasitica* and the neighboring populations have the same origin. 2) *C. parasitica* has been introduced in one of the neighboring regions and, from there, has arrived in Cantabria (or vice versa). In contrast, a study from Southern Spain showed that EU-1 was not the main VC type and made up only 6.6% of total isolates (Rodríguez-Molina et al., 2023). Therefore, the Cantabrian populations appeared similar to other North Spanish populations but not the Southern populations. Nevertheless, it was surprising that no VC type diversity was observed in the whole region as it is common to find a few VC types in European (EU) populations. One reason for no VC diversity could be the absence of sexual reproduction, as the fungus cannot reproduce sexually if a single mating type is present in a population. Sexual reproduction is a source of genetic variability, so without it, less or no genetic variability is observed. Since we found only one mating type in our study, sexual reproduction in *C. parasitica* looks unlikely in the studied area. During sampling, perithecia were not observed throughout in the studied area, which further strengthens the hypothesis of no sexual reproduction. The other reason for no VC-type diversity could be the region’s isolation, as it is a mountainous region that is comparatively isolated from the bordering areas. Within Cantabria also, the chestnut forests are rather isolated and have physical barriers between the populations, such as mountains and valleys. In other EU populations where the fungus was introduced recently, a single VC type was reported also such as Greece and North Macedonia (Sotirovski et al., 2004; Perlerou and Diamandis, 2006). Even though *C. parasitica* was present in Spain long ago, it is unknown whether it was also present in the studied populations as no information about the pathogen exists from Cantabria. The involvement of *vic* genes in other metabolic pathways (i.e., pathogenicity-related) might also be relevant to the low diversity found in northern Spain. For example, genotype EU-1 could be more pathogenic as it had significantly higher laccase activity than EU-2 (the most common VC type in Spain, Italy, Switzerland, Austria and Germany) (Robin and Heiniger, 2001; Popović et al., 2023).

A single VC type observed provides an ideal condition to apply biocontrol measures against *C. parasitica* in the region. A virus-containing (hypovirulent) strain of EU-1 type can be used as a biocontrol against the fungus. Due to the absence of a physical barrier, efficient transmission of the virus to virulent strains is likely to occur. To carry out biological control more efficiently, the proportions of virulent and hypovirulent isolates in the different regions of Cantabria should be calculated. This would save resources and biocontrol would be focussed in areas with low or no hypovirulence. As we found no VC-type diversity, the virus is expected to have no barrier in disseminating. The occurrence of the virus in the region will depend largely on if the virus is present in the region. On the other hand, a pathogenicity test of three randomly chosen isolates performed on one-year-old chestnut plants killed all the plants, showing that the isolates are highly virulent. VC-type diversity of *F. circinatum* might have provided a better comparison with *C. parasitica*. However, the information about the genetic loci of *F. circinatum* is not available so VC typing largely depends on phenotyping (Iturritxa et al., 2011).

ITS sequencing showed that *C. parasitica* is not monomorphic in the region, contrary to the anticipated lack of VC and mating type diversity. Considering the similarity between CPH-2 and CPH-3, they can be merged into a single haplogroup. The identified haplogroup exhibited a close relationship with the globally dominant haplotype, suggesting a possible common origin. In contrast, CPH-1 was closely associated with isolates reported from Eastern Europe. This could be because of the over-representation of sequences from Eastern Europe, as previously discussed (Ahmad and Baric, 2022a). Nevertheless, a different haplotype in a small region shows that at least two independent introduction events might have happened. Since all haplotypes belonged to only one VC type and one mating type, we can also hypothesize that both introductions were of the same VC type and mating type strains. In a small region of Northern Italy, similar results were found and later confirmed through microsatellite analysis (Ahmad and Baric, 2022a; Ahmad and Baric, 2022b). As *C. parasitica* is an A2-level quarantine pathogen, our results and previous studies show that the quarantine measures have not been successful, at least at the European level (Rigling and Prospero, 2018). Since the genetic diversity of the fungus is not very high in Europe, biocontrol still seems a viable option. For example, both mating types were found in Southern Spain also, but they were detected in different locations, which means sexual reproduction among them is unlikely (Rodríguez-Molina et al., 2023). Nevertheless, increasing genetic diversity could make biocontrol less efficient in European populations with high genetic diversity, such as Italy, France, and Switzerland.

Both mating types of *F. circinatum* were detected in our study. However, the presence of both mating types does not guarantee sexual reproduction because physical proximity between the mating types is required for sexual reproduction. The fact that we detected different mating types in populations far from each other means that sexual reproduction in *F. circinatum* is not likely at the moment, at least in Cantabria. However, finding opposite types in diverse populations does signify a future threat because the movement of plants within the country is not restricted and can lead to mixing mating types and promoting sexual reproduction(Vettraino et al., 2018). Therefore, we recommend limiting the movement of planting materials between different communities to decrease the sexual reproduction of the pathogen. Even though both mating types were found in our study, it is worth noting that the Cantabria region had only one mating type, so when we compare it with *C. parasitica*, we cannot say that *F. circinatum* is more diverse than *C. parasitica*. Nevertheless, the presence of two different mating types in Spain means two separate introductions of *F. circinatum* to the country as found through other genetic markers (Berbegal et al., 2013).

This is the first phylogenetic study investigating *F. circinatum* through ITS sequence analysis. Previous studies used microsatellite loci and other genetic markers to show that *F. circinatum* might be originated from North America i.e., Mexico (Wikler and Gordon, 2000; Berbegal et al., 2013). In this study, we found that the highest genetic diversity was found in Asia. The overall genetic diversity of the fungus was so low that it is hard to question its origin with the ITS sequence data alone. Nevertheless, the same haplotype found in Spain was also reported in North America. Therefore, we can confirm that the Spanish populations could have been founded by North American populations as previously established (Berbegal et al., 2013). Other populations that might be related are Asian, South African, and South American as all of them had at least one isolate of the same haplotype. In the studied area, we found no genetic diversity. A previous study utilized microsatellite markers and also found no diversity of *F. circinatum* in the region of Cantabria (Berbegal et al., 2013). Our work corroborates the last study and confirms that the ITS region is also monomorphic.

Overall, both fungi showed a low genetic diversity. In the case of *F. circinatum*, the founder effect could be responsible because the fungus was recently introduced in the country, is reproducing clonally, and did not have enough time to mutate. On the other hand, no sexual state of the fungus (*Gibberella circinata*) was found in the field in Spain. Previous studies performed in Northern Spain also showed a low genetic diversity of the fungus (Pérez-Sierra et al., 2007; Iturritxa et al., 2011; Berbegal et al., 2013). A recent paper also showed the low genetic diversity and clonality of *F. circinatum* in Spain using microsatellite markers (Fariña-Flores et al., 2023). In this study, we corroborate that the genetic diversity of the fungus continues to be low, even after many years since the previous investigations. *Cryphonectria parasitica* also had low genetic diversity in the studied area. In other Spanish populations, *C. parasitica* diversity was considerably lower than in other European populations. For example, a comparative study showed that Spain has around 2 VC types per population on average compared to Swiss populations containing approximately 10 VC types per population (Ahmad and Baric, 2022a). The Spanish VC diversity was the lowest in Europe, just higher than Greek populations. One reason for this low diversity in Spain could be natural selection pressures in the new environment that could have influenced the genetic makeup of the species over time. More research on why Spanish populations of *C. parasitica* are less diverse would help explain the ecological and environmental factors involved.

A limitation of the study is that the VC types were determined by molecular genotyping. Genotyping is very accurate and widely used, but it can only detect up to 64 types of VC (Short et al., 2015). However, at least 74 phenotypes based on VC are reported, possibly due to an additional *vic* locus or an additional allele on the known loci (Robin et al., 2000; Romon-Ochoa et al., 2021). Therefore, it should be checked if the VC-type genotype also corresponds to phenotypes by growing all cultures with VC-type tester strains. We utilized genetic markers that can provide information for biocontrol, but they are not the most suitable markers to study genetic diversity or investigate invasion histories. Hence, other molecular markers should be used to confirm these hypotheses, such as the multiple introduction events of the fungus to Cantabria. Microsatellite loci are one of the most recommended markers for studying invasion histories and comparing the overall genetic diversity of the species (Dutech et al., 2012). Such studies were already performed in Asturias (northern Spain) and pointed to a lower genetic diversity of *F. circinatum* than *C. parasitica* (3 haplotypes versus 13 haplotypes) (González-Varela et al., 2011; Fariña-Flores et al., 2023). A population genomic study could also be used to explore the role of sexual reproduction and better understand both invasive organisms in the region. A genome-scale study of *C. parasitica* showed that sexual reproduction is more frequent in Europe than previously thought using few genetic markers (Demené et al., 2019).

## Data availability statement

The original contributions presented in the study are included in the article/**Supplementary Material**, further inquiries can be directed to the corresponding author/s.

## Author contributions

FA: Formal analysis, Investigation, Methodology, Resources, Software, Visualization, Writing – original draft. JD: Conceptualization, Funding acquisition, Project administration, Supervision, Validation, Writing – review & editing.

## Supplementary material

Supplementary Table 1 The list of primers used in this study to analyse *Cryphonectria parasitica* and *Fusarium circinatum*. T_a_ refers to the annealing temperatures for each primer set.

Supplementary Table 2 Accession numbers and identities of DNA sequences (*Cryphonectria parasitica*) that were used to develop the haplotype network.

Supplementary Table 3 Accession numbers and identities of DNA sequences (*Fusarium circinatum*) that were used to develop the haplotype network.

Supplementary Data Sheet 1 A fasta alignment file of DNA sequences that was used to make the haplotype networks of *Cryphonectria parasitica*. The name of the sequence is either the haplotype name or the accession code (NCBI Genbank).

Supplementary Data Sheet 2 A fasta alignment file of DNA sequences that was used to make the haplotype networks of *Fusarium circinatum*. The name of the sequence is either the haplotype name or the accession code (NCBI Genbank).
